# Impact of depression and recreational drug use on emergency department encounters and hospital admissions among people living with HIV in Ontario: A secondary analysis using the OHTN cohort study

**DOI:** 10.1371/journal.pone.0195185

**Published:** 2018-04-09

**Authors:** Stephanie K. Y. Choi, Eleanor Boyle, John Cairney, Paul Grootendorst, Sandra Gardner, Evan J. Collins, Claire Kendall, Sean B. Rourke, for the OHTN Cohort Study

**Affiliations:** 1 Ontario HIV Treatment Network, Toronto, Ontario, Canada; 2 Faculty of Medicine, University of New South Wales, Sydney, Australia; 3 Dalla Lana School of Public Health, University of Toronto, Ontario, Canada; 4 Institute of Sports Science and Clinical Biomechanics, University of Southern Denmark, Odense, Denmark; 5 Department of Family Medicine, McMaster University, Hamilton, Ontario, Canada; 6 Institute for Clinical Evaluative Sciences, Toronto, Ontario, Canada; 7 Infant and Child Health Lab, McMaster University, Hamilton, Ontario, Canada; 8 Department of Clinical Epidemiology and Biostatistics, McMaster University, Hamilton, Ontario, Canada; 9 CanChild Centre for Childhood Disability Research, McMaster University, Hamilton, Ontario, Canada; 10 Offord Centre for Child Studies, McMaster University, Hamilton, Ontario, Canada; 11 Department of Psychiatry and Behavioural Neuroscience, McMaster University, Hamilton, Ontario, Canada; 12 Centre for Addiction and Mental Health, Toronto, Ontario, Canada; 13 Faculty of Kinesiology and Physical Education, University of Toronto, Ontario, Canada; 14 Leslie Dan Faculty of Pharmacy, University of Toronto, Ontario, Canada; 15 School of Public Policy and Governance, University of Toronto, Ontario, Canada; 16 Department of Economics, McMaster University, Hamilton, Ontario, Canada; 17 Rotman Research Institute, Baycrest Hospital, Ontario, Canada; 18 Department of Psychiatry, Faculty of Medicine, University of Toronto, Ontario, Canada; 19 University Health Network, Toronto, Ontario, Canada; 20 Bruyère Research Institute, Ottawa, Ontario, Canada; 21 University of Ottawa, Ontario, Canada; 22 The Ottawa Hospital, Ontario, Canada; 23 Centre for Urban Health Solutions, Li Ka Shing Knowledge Institute, St. Michael’s Hospital, Toronto, Ontario, Canada; Public Health Agency of Canada, CANADA

## Abstract

**Introduction:**

Nearly half of HIV-positive patients experience mental health and substance use problems, but many do not receive adequate or ongoing mental health or addiction care. This lack of ongoing care can result in the use of costly acute care services. Prospective evaluations of the relationship between psychiatric and substance use disorders and acute care services use are lacking, and this information is needed to understand unmet needs and improve access to appropriate services.

**Methods:**

We conducted a secondary data analysis from a multicenter, longitudinal, prospective cohort study (n = 3,482 adults) between October 1, 2007 and March 31, 2013. We used explanatory extended Cox proportional hazard regression models to examine the impact of current depression and recreational drug use on acute care services use, and to explore whether current depression and recreational drug use were associated with potentially avoidable acute care services use.

**Results:**

Over our 5.5 year study period, HIV-positive participants with current depression-only (aHR [95% CI]:1.2[1.1–1.4]), recreational drug use-only (1.3[1.1–1.6]), or co-occurring depression and recreational drug use (1.4[1.2–1.7]) were associated with elevated hazard of emergency department (ED) encounters compared to participants without these conditions. Over half of ED encounters were potentially avoidable. Participants with current depression-only (1.3[1.1–1.5];1.3[1.03–1.6]), recreational drug use-only (1.3[1.04–1.6];1.5[1.1–1.9]), or co-occurring depression and recreational drug use (1.3[1.04–1.7];1.4[1.06–1.9]) were associated with elevated hazard of low-acuity or repeated ED encounters respectively.

**Conclusions:**

We found a significant increase in ED services use and potentially avoidable ED encounters (including low-acuity or repeated ED encounters), particularly among those with either current depression or recreational drug use. These findings emphasize the challenges in managing HIV and mental health/addiction co-morbidities in the current HIV care model. Future research should evaluate integrated and collaborative care programs for improving the coordination of care and effectively treat mental health and addiction problems among HIV-positive patients in Ontario.

## Introduction

Nearly half of people living with HIV experience mental health and substance use problems [1–3]. However, many of these individuals, particularly those who are the most socially disadvantaged, do not receive adequate mental health or addiction care and support [4–7]. Suboptimal mental health and addiction treatment for those with HIV increases the probability of non-adherence to combination antiretroviral therapy (cART), and the risk of poorer clinical outcomes and co-morbidity and mortality. Suboptimal mental health and addiction care may also result in the increased use of costly acute care services, such as emergency department (ED) encounters and hospital admissions [4, 8–12].

A large body of literature has described the impacts of mental health and substance use-related problems on acute care services use among patients with multi-morbidities [13, 14]. For example, in a recent meta-analysis, Dickens et al. (2012) found that patients with chronic health conditions and depression had a 49% higher likelihood of using inpatient services compared to patients without depression [14]. A recent scoping review also demonstrated that mental health disorders and substance use problems are important factors for frequent emergency department use and the unscheduled use of health services among patients with long-term chronic conditions [13].

Similar patterns of acute care use have been observed among people living with HIV. In the last several decades, the overall rate of hospitalization among people living with HIV on combination antiretroviral therapy has declined, but their rate of hospital admissions for concomitant psychiatric disorders and substance use has increased [9, 12, 15]. Two U.S. studies found that hospitalization for psychiatric disorders among people living with HIV increased by 42–73% from the mid-1990s to the early 2000s [9, 12]. A recent meta-analysis of 85 cohort studies (with data published after 2007) reported that psychiatric disorders were one of the most common non-AIDS-related diagnoses for hospital admissions in Europe among those with HIV, based solely on pooled proportions of key diagnoses in hospital admissions [15]. With respect to ED encounters, Josephs et al. (2010) found that HIV-positive illicit drug users (current or former) in the United States had a 59–85% higher rate of ED encounters compared to those who never used drugs [10]. In another recent U.S. study of people living with HIV who presented to the ED, those with mental health and substance use problems had a higher rate of subsequent hospital admissions, admissions to other facilities, or discharge against medical advice [8].

However, very few studies have assessed the prospective relationship between co-occurring psychiatric and substance use disorders and acute care services use among people living with HIV. Most of the current studies have been limited by cross-sectional or ecological designs, or by self-reported acute care services use. Additionally, most studies have been from the United States or Europe. The sole Canadian study was limited to injection drug users and focused on general ED encounters only. This latter study also controlled for only a limited number of social demographics, and did not consider co-existing psychiatric disorders [11].

Given these limitations, the primary goal of our study was to determine the relationship of current depression and recreational drug use on acute care. Our second goal was to determine how current depression and recreational drug use were associated with potentially avoidable acute care use. Understanding these relationships is critical in resource-constrained health care systems for planning and designing cost-effective preventive and responsive mental health care programs for people living with HIV.

## Materials and methods

### Study design

We analysed data collected between October 1, 2007 and March 31, 2013 using linked data from the population-based administrative health databases held at the Institute for Clinical Evaluative Sciences (ICES) and the Ontario HIV Treatment Network Cohort Study (OCS). The study was approved by the HIV research ethics board of the University of Toronto and the institutional review boards at Sunnybrook Health Sciences Centre and participating HIV clinics across Ontario (Ottawa Health Science Network Research Ethics Board, University of Western Ontario Research Ethics Board, St. Michael's Hospital Research Ethics Board, the Research Ethics Board of Health Sciences North, Sunnybrook Health Sciences Centre Research Ethics Board, University Health Network Research Ethics Board, and Windsor Regional Hospital Research Ethics Board).

### Data sources

The OCS is a longitudinal and multicentre prospective cohort of HIV-positive participants, and contains a wide range of socio-demographic information, psychosocial and behavioural data, and medical and clinical data. A full description of the OCS cohort has been published elsewhere [16]. ICES holds a comprehensive collection of administrative claims and billing data in the province of Ontario. These two de-identified databases were linked at the individual level using encrypted health care numbers as unique identifiers and were made available through a data sharing agreement with the Ontario Ministry of Health and Long Term Care. Databases used for this study and held by ICES were: the Ontario Health Insurance Plan (OHIP) database, the Canadian Institute for Health Information discharge abstract database (DAD), the National Ambulatory Care Reporting System (NACRS), and the Registered Persons Database (RPDB).

These databases contain all medical and hospital services covered by OHIP (a province-wide publicly-funded insurance plan) for all residents of Ontario. The OHIP claims database captures most physician claim billings in the province. NACRS contains all ED encounters and information regarding their triage level, diagnosis at discharge, and visit disposition at discharge from the ED [17]. DAD contains abstracts of all discharges from acute, chronic, and rehabilitation inpatient facilities [18]. The RPDB, which contains Ontario vital statistics data, was used to obtain OCS participants’ dates of death [19]. Details about the linked data source have been provided in recent studies [5, 20].

### Study population

OCS participants were included if they were eligible for OHIP and had completed their first standard OCS questionnaire on or prior to March 31, 2012, allowing for at least one year to observe acute care services use events. The date of completion of the first questionnaire served as the baseline date. We excluded 13 OCS participants who had missing measures on current depression and recreational drug use at the baseline visit. We further excluded 206 OCS participants who had at least one ED encounter or hospital admission 30 days prior to baseline to ensure that the participants were not re-admitted for a problem that was identified prior to baseline. We included 3,482 OCS participants in our final analyses. The median number of days between interviews was 372 (interquartile range: 339, 452 days). The median length of follow-up was 2.8 years (interquartile range: 1.2, 3.6 years). The median number of interviews completed was 3 (interquartile range: 2, 4).

### Main Exposure: Current depression and recreational drug use

The Center for Epidemiologic Studies Depression (CES-D_20_) scale with a cut point of 23 (Sensitivity [SE]: 1.0; Specificity [SP]: 0.87) or the Kessler Psychological Distress Scale (K_10_) scale with a cut point of 22 (SE: 0.97, SP: 0.81) was used to identify “current depression” during the past four weeks. Current depression and recreational drug use status were measured at baseline and at each follow-up interview during the five-year study period. As described in recent articles, due to resource-constraints at several participating HIV clinics, K_10_ was administered to 61% of OCS participants and the CES-D_20_ was administered to the rest [5, 20]. The excellent reliability and diagnostic accuracy of these two screening instruments have been validated in a sample of OCS participants when compared to the Diagnostic and Statistical Manual of Mental Disorders, 4th Edition, Text Revision (DSM-IV-TR) criteria for major depression [21]. Additionally, the CES-D_20_ and K_10_ demonstrate good inter-rater agreement (Cohen’s Kappa Statistic = 0.79) when compared to the DSM-IV-TR criteria for a diagnosis of major depression [21].

In the interview, OCS participants were asked whether they had used any of the following drugs for recreational or other non-medical purposes over the past six months: anabolic steroids, amphetamines, methamphetamines, cocaine, crack/freebase, club drugs, heroin, opiates, tranquilizers, or other substances. We did not include alcohol use as a measure of recreational drug use because our linked dataset does not have a reliable measure for this condition.

We then categorized our main exposure variable into four groups: (1) those with current depression only; (2) those with recreational drug use only; (3) those with co-occurring current depression and recreational drug use; and (4) those without either current depression or recreational drug use. This variable was updated at each OCS interview.

### Primary and secondary outcomes: ED encounters, hospital admissions, and potentially avoidable ED encounters

Our primary outcomes were the index ED encounters and hospital admissions during our study period. We included hospital admissions with at least one overnight stay. We excluded visits related to delivery of a baby and its complications (International Classification of Diseases, 10th Revision).

Among OCS participants who had at least one ED encounter, we further examined three types of potentially avoidable ED encounters as secondary outcomes.

1. *ED encounters with low-acuity* were identified using level four or five of the five-level Canadian triage and acuity scale (1 = resuscitation, 2 = emergency, 3 = urgent, 4 = semi-urgent, 5 = non-urgent) AND a visit disposition at discharge that indicated the participant was not transferred to inpatient care [22].
2. *Repeated ED encounters* were defined by the discharge date of an ED encounter that occurred 30 days following the index encounter at the same or a different facility [23].
3. *ED encounters without seeking ambulatory care* were defined as an ED encounter without the participant having sought ambulatory care 30 days prior to the index encounter [24].

### Covariates

We attempted to control for the effects of covariates that may be associated with current depression and recreational drug use that might be associated with the use of acute care services among OCS participants. These covariates included “need,” “predisposing,” and “enabling” factors as defined in Andersen et al.’s socio-behavioural theoretical framework for health services use [25].

*Need factors* are OCS participants’ perceived and evaluated health status. For the purposes of our study, they included: the use of antidepressants (identified by the OCS participants’ medical records); CD4 cell counts of less than 200 μL (yes/no; based on HIV antigen test records during the past six months); non-suppressed viral loads (>50 μL) (yes/no; based on HIV viral load records during the past six months); number of years since HIV diagnosis; current smoking (yes/no); physical health-related quality of life (based on the 12-Item Short Form Survey, 2^nd^ version); current combination antiretroviral therapy use (yes/no; based on OCS participants’ medical records); and physical multi-morbidity based on the Charlson-Deyo multi-morbidity index of 17 ICD physical disease categories. We also included history of depression (OHIP-ICD: 296 or 311), history of drug addiction/dependence (OHIP-ICD: 304), and history of alcoholism (OHIP-ICD: 303), which were determined by whether the OCS participants had a relevant diagnostic code in the OHIP database from the earliest available records in these databases to a year before baseline (allowing a year as a washout period to avoid overlap of current and past depression and substance use diagnosis).

*Predisposing factors* are characteristics that may predispose a person to seek care. We included the following predisposing factors: age (grouped as 16–29, 30–39, 40–49, and ≥50 years); self-reported gender (female vs. male); sexual orientation (self-reported as lesbian, gay, bisexual or heterosexual); relationship status (married or living with a partner; or single, separated, divorced, or widowed); self-reported ethnicity (grouped as Aboriginal, African/Caribbean/Asian/Latin American, or European decent); immigration status (grouped as Canadian immigrant or Canadian born); current employment status (categorized as employed, unemployed, student/retired, or recipient of Ontario Disability Support Program [ODSP]), and educational attainment (completed high school or less vs. more than high school education).

*Enabling factors* are resource-related characteristics that may enable OCS participants to seek care. We included the following enabling factors: annual household income levels before taxes and benefits (grouped as <$20,000, $20,000–$39,999, $40,000–$49,999, or ≥$50,000 CAD); perceived difficulty in affording housing-related expenses; and perception of housing and neighbourhood conditions.

Each of these covariates (except for the Charlson-Deyo multi-morbidity index, history of depression, history of alcoholism, and history of drug addiction/dependence) were time-varying and updated at each OCS interview.

### Statistical analysis

All statistical analyses were two-sided, had an a priori statistical significance level of p <0.05, and were performed using STATA MP v. 13.1 [26]. All analyses were conducted at an ICES secured computing centre.

We estimated the incidence rates (per 100 person-years) of ED encounters and hospital admissions during our 5.5-year study period of the whole cohort and by their depression and recreational drug use status reported at the baseline. Among those who had at least one ED admission, we further estimated the incidence rates of low-acuity and repeated ED admission and ED admission without seeking ambulatory care.

We constructed two explanatory extended Cox proportional hazard regression models to examine the association between time-varying current depression and recreational drug use status and index (1) ED encounters, and (2) hospital admissions during the 5.5-year follow-up period among OCS participants. There were 43 deaths in our study cohort during the five-year period, and most occurred after the index ED encounters or hospital admissions. For this reason, we censored the participants at their date of death.

Among those who had at least one ED encounter, we constructed three additional explanatory extended Cox proportional hazard regression models to examine the impact of time-varying current depression and recreational drug use status on the index: (1) low-acuity ED encounters; (2) repeated ED encounters; and (3) ED encounters without seeking ambulatory care 30 days prior to the index encounter.

When selecting covariates in each multivariable regression model, we adopted a method similar to a recent study [20]. Briefly, we used backward selection method [27–29]. At each step of the backward selection procedure, four p-value thresholds (p< 0.004211, p< 0.15, p< 0.1, or p< 0.2) were considered separately to determine whether a covariate should be retained in the model. The p-value threshold was calculated by Akaike information criteria [AICs] or Bayesian information criteria [BICs] of two nested models [27–29]. Additionally, in each backward selection procedure, we forced covariates in the multivariable models based on a priori evidence from the literature [8–12, 15]. We forced gender, perceived difficulty in affording housing-related expenses, and current employment status in the multivariable models for ED and potentially avoidable ED encounters. We forced age, CD4 cell counts of less than 200 μL, non-suppressed viral loads, self-reported ethnicity, current employment status, multi-morbidity index, and physical quality-of-life in multivariable models for hospital admissions, The final set of covariates was selected based on the lowest AICs and BICs of the full model (including all covariates) and the final models according to the four p-value criteria [27–29]. We further examined the uncertainty of this model building procedure by generating 1,000 bootstrapped samples from the original dataset to examine the probability of each covariate (without a priori evidence) included in the model [27–29].

For each final explanatory multivariable model, we controlled for differences between the CES-D_20_ and K_10_ as well as the residual difference of OCS participants’ characteristics by clinic type [20]. In addition, we tested proportionality assumptions in each final model using Schoenfeld residuals against time for each of the covariates and interactions with linear time and natural log of time [27, 29]. When a covariate did not meet the proportionality assumptions, we included an interaction term between any covariate and natural log of time in each final model.

Adjusted hazard ratios (aHRs) and 95% confidence intervals (CI) were reported.

## Results

Over the 5.5-year study period, we included 3,482 OCS participants at baseline with a median 2.3 years of follow-up (95% CI: 2.2–2.5). The median age of OCS participants was 46 years (ranging from 17 to 85 years), and 574 (16.5%) were female. Of the cohort, 696 (20.0%) were identified as having current depression only, 396 (11.4%) with recreational drug use only, 253 (7.3%) with co-occurring current depression and recreational drug use, and 2,137 (61.4%) with no current depression or recreational drug use (see Table 1).

**Table 1:** Baseline characteristics by current depression and recreational drug use status (N = 3,482).

Characteristics	Current depression (CD) and recreational drug use (RDU) status			
	Without CD & RDU*	With CD-only	With RDU-only	Co-occurring CD & RDU
	(N = 2137, 61.4%)	(N = 696, 20.0%)	(N = 396, 11.4%)	(N = 253, 7.3%)
**Demographic characteristics**				
Age				
16–29 years	110 (5.1%)	42 (6.0%)	48 (12.1%)	22 (8.7%)
30–39 years	343 (16.1%)	143 (20.5%)	82 (20.7%)	67 (26.5%)
40–49 years	86 6(40.5%)	306 (44.0%)	178 (44.9%)	120 (47.4%)
≥ 50 years	818 (38.3%)	205 (29.5%)	88 (22.2%)	44 (17.4%)
Gender				
Female	349 (16.3%)	159 (22.8%)	28 (7.1%)	38 (15.0%)
Male	1787 (83.6%)	537 (77.2%)	368 (92.9%)	215 (85.0%)
Sexual orientation				
Gay, lesbian, or bisexual	1420 (66.4%)	422 (60.6%)	333 (84.1%)	168 (66.4%)
Heterosexual	704 (32.9%)	270 (38.8%)	63 (15.9%)	83 (33.8%)
Marital status				
Married / living with partner	945 (44.2%)	232 (33.3%)	144 (36.4%)	63 (24.9%)
Single, separated/divorced, or widowed	1189 (55.6%)	462 (66.4%)	251 (63.4%)	185 (73.1%)
Ethnicity				
Aboriginal	177 (8.3%)	80 (11.5%)	38 (9.6%)	44 (17.4%)
African, Caribbean, Asian or Latin American	497 (23.3%)	176 (25.3%)	55 (13.9%)	29 (11.5%)
European decent	1457 (68.2%)	437 (62.8%)	301 (76.0%)	180 (71.1%)
Immigration status				
Canadian immigrant	667 (31.2%)	232 (33.3%)	74 (18.7%)	43 (17.0%)
Canadian born	1465 (68.6%)	463 (66.5%)	321 (81.1%)	209 (82.6%)
**Socio-economic Status**				
Employment status				
Unemployed	166 (7.8%)	82 (11.8%)	41 (10.4%)	35 (13.8%)
Student/retired	241 (11.3%)	37 (5.3%)	23 (5.3%)	7 (2.8%)
Recipient of Ontario Disability Support Program	541 (25.3%)	380 (54.6%)	112 (28.3%)	143 (56.5%)
Employed	1185 (55.5%)	193 (27.7%)	219 (55.3%)	67 (26.5%)
Educational attainment				
Completed high school or less	627 (29.3%)	284 (40.8%)	100 (25.3%)	108 (42.7%)
Completed more than high school	1510 (70.7%)	412 (59.2%)	296 (74.7%)	145 (57.3%)
Annual household income (CAD) before withholding taxes/benefits				
< $20,000	433 (20.3%)	263 (37.8%)	82 (20.7%)	111 (43.9%)
$20,000 to $39,999	371 (17.4%)	160 (23.0%)	72 (18.2%)	37 (14.6%)
$40,000 to $49,999	323 (15.1%)	63 (9.1%)	40 (10.1%)	20 (7.9%)
≥ $50,000	829 (38.8%)	136 (19.5%)	162 (40.9%)	51 (20.2%)
** Housing and Neighbourhood Conditions**				
Difficulty in affording housing-related expenses ^a^				
Yes	376 (17.6%)	264 (37.9%)	71 (17.9%)	83 (32.8%)
No	1761 (82.4%)	432 (62.1%)	325 (82.1%)	170 (67.2%)
Worry about eviction ^b^				
Yes	235 (11.0%)	182 (26.1%)	45 (11.4%)	60 (23.7%)
No	1901 (89.0%)	514 (73.9%)	351 (88.6%)	193 (76.3%)
Control over housing situation ^b^				
Yes	1732 (81.0%)	438 (62.9%)	317 (80.1%)	164 (64.8%)
No	405 (19.0%)	258 (37.1%)	79 (19.9%)	89 (35.2%)
Sense of belonging to neighbourhood ^b^				
Yes	1533 (71.7%)	372 (53.4%)	273 (68.9%)	136 (53.8%)
No	604 (28.3%)	324 (46.6%)	123 (31.1%)	117 (46.2%)
Perceived good location of home ^b^				
Yes	1725 (80.7%)	450 (64.7%)	324 (81.8%)	164 (64.8%)
No	412 (19.3%)	246 (35.3%)	72 (18.2%)	89 (35.2%)
** Health Status**				
History of depression ^c^				
Yes	673 (31.5%)	384 (55.2%)	153 (38.6%)	157 (62.1%)
No	1464 (68.5%)	312 (44.8%)	243 (61.4%)	96 (37.9%)
History of alcoholism ^d^				
Yes	152 (7.1%)	92 (13.2%)	65 (16.4%)	68 (26.9%)
No	1962 (91.8%)	596 (85.6%)	330 (83.3%)	184 (72.7%)
History of drug addiction/dependence ^e^				
Yes	308 (14.4%)	149 (21.4%)	126 (31.8%)	126 (49.8%)
No	1806 (84.5%)	539 (77.4%)	269 (67.9%)	126 (49.8%)
Current smoker				
Yes	683 (32.0%)	325 (46.7%)	217 (54.8%)	176 (69.6%)
No	1450 (67.9%)	369 (53.0%)	177 (44.7%)	76 (30.0%)
Current use of combination antiretroviral Therapy				
Yes	1865 (87.3%)	594 (85.3%)	313 (79.0%)	188 (74.3%)
No	272 (12.7%)	102 (14.7%)	83 (21.0%)	65 (25.7%)
Antidepressant use ^f^				
Yes	374 (17.5%)	284 (40.8%)	89 (22.5%)	108 (42.7%)
No	1763 (82.5%)	412 (59.2%)	307 (77.5%)	145 (57.3%)
Charlson physical multi-morbidity index ≥ 1				
Yes	491 (23.0%)	206 (29.6%)	78 (19.7%)	80 (31.6%)
No	1646 (77.0%)	490 (70.4%)	318 (80.3%)	173 (68.4%)
Recent CD4 cell count (<200 *μL*)				
Yes	190 (8.9%)	70 (10.1%)	44 (11.1%)	43 (17.0%)
No	1944 (91.0%)	625 (89.8%)	352 (88.9%)	209 (82.6%)
Detectable recent viral load (>50 *μL*)				
Yes	493 (23.1%)	191 (27.4%)	138 (34.8%)	126 (49.8%)
No	1644 (76.9%)	505 (72.6%)	258 (65.2%)	127 (50.2%)
Years since HIV diagnosis, median (25^th^ to 75^th^ percentile)	11.7 (5.8–17.7)	12.0 (5.4–16.8)	8.8 (3.4–15.7)	7.7 (3.2–14.8)
Physical quality-of-life (SF12v2), median (25^th^ to 75^th^ percentile)	53.2 (45.4–56.7)	46.0 (36.4–55.2)	54.8 (47.8–7.8)	46.2 (37.5–56.8)
**Instrument Type** ^g^				
CES-D_20_	897 (42.0%)	242 (34.8%)	148 (37.4%)	54 (21.3%)
K_10_	1240 (58.0%)	454 (65.2%)	248 (62.6%)	199 (78.7%)

* The reference group is OCS participants without current depression and/or recreational drug use.

^a^ Difficulty in affording house-related expenses was defined as a patient’s self-reported “Very difficult” or “Fairly difficult” to the following question: *“Considering your household income*, *how difficult is it for you to meet your monthly housing-related costs*?*(Housing costs include rent/mortgage*, *property taxes and utilities only)*.*”*

^b^ A 5-point Likert scale (strongly agree to strongly disagree) was used. We dichotomized their response into “yes” (strongly agree/agree) and “no” (neutral/disagree/strongly disagree).

^c^ History of depression was defined as having a past depression-related diagnosis in OHIP records (OHIP ICD-9: 296 and 311), from the earliest available records to a year before baseline.

^d^ History of alcoholism was defined as a diagnostic code of alcohol dependence/abuse in OHIP (ICD-9: 303) from the earliest available records to a year before baseline.

^e^ History of drug addiction/dependence was defined as a diagnostic code of drug dependence/addiction in OHIP (ICD-9: 304) from the earliest available records to a year before baseline.

^f^ The definition of antidepressants was based on the first line of antidepressants for managing depression in adults recommended by the Canadian Network for Mood and Anxiety Treatments (CANMAT) Clinical guidelines (Lam et al., 2009).

^g^ There are two instruments for identifying current depression administered by clinic nurses and assistants during the participant’s regular clinical appointments. Due to constraints on human resources and time in several HIV clinics, 61% of HIV-positive participants were administered the 10-item Kessler Psychological Distress Scale (K_10_) and 39% were administrated the 20-item Centre for Epidemiologic Studies Depression Scale (CES-D_20_). Full details of the cohort can be found on the study website: http://www.ohtncohortstudy.ca/

Table 1 depicts the baseline characteristics by current depression and recreational drug use status. When compared to OCS participants with neither current depression nor recreational drug use, those with co-occurring conditions were more likely to be younger (<50 years) and more likely to be Canadian born. In addition, OCS participants with co-occurring conditions were more likely to live in vulnerable situations. For example, they were more likely to be unemployed or receiving ODSP, have basic education only, have a low household income, and feel dissatisfied with their home or neighborhood. OCS participants with co-occurring conditions were also more likely to have a history of multi-morbidity, depression, alcoholism, and drug addiction, and were more likely to be current tobacco users. They were also more likely to have detectable viral loads and less likely to be currently taking cART.

Similar patterns of characteristics were found among OCS participants in the current depression-only subgroup. However, these participants were more likely to be female and heterosexual compared to participants without current depression or recreational drug use.

For the recreational drug use-only subgroup, participants were more likely to be male, and gay or bisexual. However, we did not find any statistically significant differences among these participants in terms of employment status, annual household incomes, histories of multi-morbidity, or housing satisfaction compared to OCS participants with neither current depression nor recreational drug use.

### Crude incidence rate of acute care services use

Over the 5.5-year study period, of the 3,482 participants, there were 25.6 per 100-years (95% CI: 24.5–26.8) and 6.9 per 100 person-years (95% CI: 6.4–7.4) incidence of ED encounters and hospital admissions respectively. The current depression and recreational drug use status were based on the baseline line measure. OCS participants with either current depression or recreational drug use had about a 1.3–2.2 fold higher incidence of ED encounters and hospital admissions compared to those without these exposures (Table 2). We did not find that OCS participants in the recreational drug use-only subgroup had a higher incidence of hospital admissions compared to those without either current depression or recreational drug use.

**Table 2:** Crude incidence rate (IR) per 100 person-years, crude hazard ratio (HR), and 95% confidence intervals (CI) for emergency department (ED) encounters, hospital admissions, and potentially avoidable ED encounters, whole cohort and by current depression and recreational drug use status measured at the baseline.

	ED encounters		Hospital admissions		Potentially avoidable ED encounters					
					(N = 2,108)					
	(N = 3,482)		(N = 3,482)		Low-acuity ED encounters ^a^		Repeated ED encounters ^b^		ED encounters without seeking ambulatory care ^c^	
	IR	Crude HR Ratio	IR	Crude HR Ratio	IR	Crude HR Ratio	IR	Crude HR Ratio	IR	Crude HR Ratio
	(95% CI)	(95% CI)	(95% CI)	(95% CI)	(95% CI)	(95% CI)	(95% CI)	(95% CI)	(95% CI)	(95% CI)
**Cohort**	25.6		6.9		19.4		10.9		7.9	
	(24.5, 26.8)		(6.4, 7.4)		(18.3, 20.6)		(10.2, 11.7)		(7.2, 8.6)	
**Current depression and recreational drug use**										
Current depression-only	34.8	**1.6**	9.1	**1.5**	21.8	**1.2**	12.2	**1.3**	7.3	1.03
	(31.8, 38.0)	**(1.4, 1.7)**	(7.9, 10.4)	**(1.3, 1.8)**	(19.4, 24.6)	**(1.1, 1.4)**	(10.5, 14.1)	**(1.1, 1.5)**	(6.5, 8.2)	(0.8, 1.3)
Recreational drug use-only	30.0	**1.3**	6.0	1.0	22.8	**1.3**	13.2	**1.4**	7.5	**1.5**
	(26.4, 34.1)	**(1.2, 1.5)**	(4.8, 7.6)	(0.8, 1.3)	(19.2, 27.0)	**(1.1, 1.6)**	(10.8, 16.2)	**(1.1, 1.8)**	(6.3, 9.0)	**(1.2, 1.9)**
Co-occurring current depressive symptoms and recreational drug use	51.9	**2.2**	13.0	**2.2**	25.6	**1.4**	16.9	**1.8**	10.8	1.2
	(44.8, 60.1)	**(1.9, 2.6)**	(10.4, 16.1)	**(1.7, 2.7)**	(21.1, 31.1)	**(1.1, 1.7)**	(13.5, 21.1)	**(1.4, 2.3)**	(8.7, 13.4)	(0.9, 1.7)
Without current depression and recreational drug use (reference)	21.2	1	5.9	1	17.2	1	9.4	1	9.2	1
	(20.0, 22.4)		(5.4, 6.5)		(15.8, 18.6)		(8.5, 10.3)		(6.9, 12.2)	

IR = Incidence rate

HR = Hazard ratio

CI = Confidence intervals

ED = Emergency department encounters

^a^ Low-acuity ED encounters were identified using level four or five of the five-level Canadian triage and acuity scale (1 = resuscitation, 2 = emergency, 3 = urgent, 4 = semi-urgent, 5 = non-urgent) AND a visit disposition at discharge that indicated the participants were not transferred to inpatient care.

^b^ Repeated ED encounters were defined by discharge date of an emergency department encounter that occurred within 30 days of a previous encounter at the same or a different facility.

^c^ Emergency department encounters without seeking ambulatory care were defined as emergency department encounters without an ambulatory visit to a physician in the 30 days prior to the index encounter

Among the 2,108 OCS participants with at least one ED encounter, there were 19.4 per 100-years (95% CI: 18.3–20.6), 10.9 per 100 person-years (95% CI: 10.2–11.7), and 7.9 per 100 person-years (95% CI: 7.2–8.6) incidence of low-acuity ED encounters, repeated ED encounters, and ED encounters without seeking ambulatory care, respectively. Based on the current depression and recreational drug use status at the baseline, OCS participants with either current depression or recreational drug use had about a 1.2–1.8 fold higher incidence of low-acuity ED encounters or repeated ED encounters compared to those without either current depression or recreational drug use. In addition, OCS participants in the recreational drug use-only subgroup had a 1.5 fold higher incidence of having an ED encounter without having sought ambulatory care 30 days prior to the index encounter (compared to those with neither current depression nor recreational drug use problems).

### Independent associations between current depression and/or recreational drug use main exposures on acute care services use

Table 3 demonstrates the multivariable associations between participant characteristics and ED encounters and hospital admissions. After adjustment, compared to OCS participants with neither current depression nor recreational drug use, those with current depression only (aHR: 1.2; 95% CI: 1.1–1.4), recreational drug use only (aHR: 1.3; 95% CI: 1.1–1.6), or co-occurring conditions (aHR: 1.4; 95% CI: 1.2–1.7) were associated with elevated hazard of ED encounters. For hospital admission, after adjustment, OCS participants with current depression-only (aHR: 1.2; 95% CI: 1.1–1.5) or co-occurring conditions (aHR: 1.6; 95% CI: 1.2–2.1) were associated with elevated hazard of hospital admissions compared to OCS participants without these conditions.

**Table 3:** Crude and adjusted hazard ratio (aHR) with 95% confidence intervals (CI) for associations between depression and recreational drug use exposure and two acute care services use outcomes (N = 3,482).

Main Exposure	Emergency Department Encounters		Hospital Admissions	
	Crude HR	aHR	Crude HR	aHR
	(95% CI)	(95% CI)	(95% CI)	(95% CI)
Depression-only^a^	**1.6**	**1.2**	**1.5**	**1.3**
	**(1.4, 1.7)**	**(1.06, 1.6)**	**(1.3, 1.8)**	**(1.06, 1.53)**
Recreational Drug Use—only^b^	**1.3**	**1.3**	1.0	1.1
	**(1.2, 1.5)**	**(1.1, 1.6)**	(0.8, 1.3)	(0.9, 1.5)
Co-occurring depression and recreational drug use	**2.2**	**1.4**	**2.2**	**1.6**
	**(1.9, 2.6)**	**(1.2, 1.7)**	**(1.7, 2.7)**	**(1.2, 2.1)**
Without depression and recreational drug use(reference)	1	**1**	1	**1**

This table contains the final set of covariates retained in the multivariable Cox proportional hazard regression models for the index emergency department encounters and hospital admissions. More information about the full model can be found in S1 Table.

aHR = Adjusted hazard ratios

CI = Confidence intervals

^a^ There are two instruments for identifying current depression administered by clinic nurses and assistants during the participant’s regular clinical appointments. Due to constraints on human resources and time in several HIV clinics, 61% of HIV-positive participants were administered the 10-item Kessler Psychological Distress Scale (K_10_) and 39% were administrated the 20-item Centre for Epidemiologic Studies Depression Scale (CES-D_20_). Full details of the cohort can be found on the study website: http://www.ohtncohortstudy.ca/

^b^ Participants were asked whether they had used any of the following drugs for recreational or other non-medical purposes over the past six months: anabolic steroids, amphetamines, methamphetamines, cocaine, crack/freebase, club drugs, heroin, opiates, tranquilizers, or other substances.

Table 4 demonstrates the multivariable associations between participant characteristics and the three types of potentially avoidable ED encounters. Among 2,108 OCS participants with at least one ED encounter, our multivariable analysis demonstrated that OCS participants with current depression-only (aHR: 1.3; 95% CI: 1.1–1.5), recreational drug use-only (aHR: 1.3; 95% CI: 1.04–1.6), or co-occurring conditions (aHR: 1.3; 95% CI: 1.04–1.7) were associated with elevated hazard of low-acuity ED encounters compared to OCS participants without current depression and recreational drug use. Additionally, OCS participants with depression only (aHR: 1.3; 95% CI: 1.03–1.6), recreational drug use only (aHR: 1.5; 95% CI: 1.1–1.9), or co-occurring conditions (aHR: 1.4; 95% CI: 1.1–1.9) were associated elevated hazard of repeated ED encounters. Furthermore, OCS participants with recreational drug use only (aHR: 1.5; 95% CI: 1.2–2.0) were associated with elevatedhazard of ED encounters without having sought ambulatory care 30 days prior to the index encounter.

**Table 4:** Adjusted hazard ratio (aHR) with 95% confidence intervals (CI) for associations between current depression and recreational drug use exposure and three potentially avoidable emergency department (ED) encounter outcomes (N = 2,108).

Main Exposure	Low-acuity ED Encounters ^a^		Repeated ED Encounters ^b^		ED encounters without seeking ambulatory care 30 days prior to the index encounters ^c^	
	Crude HR	aHR	Crude HR	aHR	Crude HR	aHR
	(95% CI)	(95% CI)	(95% CI)	(95% CI)	(95% CI)	(95% CI)
Depression-only ^d^	**1.2**	**1.2**	**1.3**	**1.3**	1.03	0.97
	**(1.1, 1.4)**	**(1.1, 1.5)**	**(1.1, 1.5)**	**(1.03, 1.6)**	(0.8, 1.3)	(0.8, 1.3)
Recreational drug use -only ^e^	**1.3**	**1.3**	**1.4**	**1.5**	**1.5**	**1.5**
	**(1.1, 1.6)**	**(1.04, 1.6)**	**(1.1, 1.8)**	**(1.1, 1.9)**	**(1.2, 1.9)**	**(1.2, 2.0)**
Co-occurring depression and recreational drug use	**1.4**	**1.3**	**1.8**	**1.4**	1.2	0.9
	**(1.1, 1.7)**	**(1.04, 1.7)**	**(1.4, 2.3)**	**(1.1, 1.9)**	(0.9, 1.7)	(0.7, 1.4)
Without depression and recreational drug use (reference)	1		1		1	**1**

This table contains the final set of covariates retained in the multivariable Cox proportional hazard regression models for the three potentially avoidable emergency department encounter outcomes. More information about the full model can be found in S2 Table.

aHR = Adjusted hazard ratios

CI = Confidence intervals

^a^ Low-acuity ED encounters were identified using level four or five of the five-level Canadian triage and acuity scale (1 = resuscitation, 2 = emergency, 3 = urgent, 4 = semi-urgent, 5 = non-urgent) AND a visit disposition at discharge that indicated that participants were not transferred to inpatient care.

^b^ Repeated ED encounters were defined by discharge date of an emergency department encounter that occurred within 30 days of a previous encounter at the same or a different facility.

^c^ Emergency department encounters without seeking ambulatory care were defined as emergency department encounters without an ambulatory visit to a physician in the 30 days prior to the index encounter.

^d^ There are two instruments for identifying current depression administered by clinic nurses and assistants during the participant’s regular clinical appointments. Due to constraints on human resources and time in several HIV clinics, 61% of HIV-positive participants were administered the 10-item Kessler Psychological Distress Scale (K_10_) and 39% were administrated the 20-item Centre for Epidemiologic Studies Depression Scale (CES-D_20_). Full details of the cohort can be found on the study website: http://www.ohtncohortstudy.ca/

^e^ Participants were asked whether they had used any of the following drugs for recreational or other non-medical purposes over the past six months: anabolic steroids, amphetamines, methamphetamines, cocaine, crack/freebase, club drugs, heroin, opiates, tranquilizers, or other substances

## Discussion

This is the first study to examine the relative impacts of current depression and recreational drug use on the incidence of acute care services use among HIV-positive participants in Canada. We found that HIV-positive participants with current depression-only (aHR: 1.2; 95% CI: 1.1–1.4), recreational drug use-only (aHR: 1.3; 95% CI: 1.1–1.6), or co-occurring conditions (aHR: 1.4; 95% CI: 1.2–1.7) were associated with a higher hazard of ED encounters compared to those without these conditions. Those with current depression only (aHR: 1.2; 95% CI: 1.1–1.5) or co-occurring current depression and recreational drug use conditions (aHR: 1.6; 95% CI: 1.2–2.1) were associated with elevated hazard of hospital admission. More importantly, over half of these ED encounters were potentially avoidable (low-acuity or repeated ED encounters, or ED encounters without seeking ambulatory care). Participants with current depression-only (1.3[1.1–1.5]; 1.3[1.03–1.6]), recreational drug use-only (1.3[1.04–1.6]; 1.5[1.1–1.9]), or co-occurring depression and recreational drug use (1.3[1.04–1.7]; 1.4[1.06–1.9]) were associated with elevated hazard of low-acuity or repeated ED encounters respectively. The association of current depression and/or recreational drug use with ED encounters is consistent with prior studies. For example, two recent meta-analyses showed that current depression was associated with more acute care services utilization in the general population (pooled estimate: 1.36) [30] and among patients with chronic physical conditions (pooled estimate: 1.49) [14]. Among HIV-positive participants, our results are comparable to the findings of the two cross-sectional studies conducted in the U.S. reporting that the co-occurrence of severe mental health disorders and injection drug use was associated with greater hospital admissions [31] and that recreational drug use was associated with higher odds of presentation to the ED [10]. Our cumulative incidence of ED use was also comparable to the estimate reported in a sample of 428 HIV-positive injection drug users in British Columbia, Canada (61% vs. 63%) [11].

Notably, our findings offer new information regarding the potentially avoidable context of acute care use among people living with HIV and concurrent depression and recreational drug use. There are several potential reasons for our findings. First, untreated depression and substance use problems can create barriers for people living with HIV to engage in and maintain regular HIV care. Depression is significantly associated with a lower likelihood of achieving optimal adherence to cART in low-, middle-, and high-income countries [32]. It is possible that patients with unstable psychosocial situations are hesitant to initiate cART because of their fear of drug resistance resulting from missed doses [33]. The resultant non-adherence to cART might also increase the likelihood of ED encounters. Second, in North America, disparities in the delivery of mental health care persist: over half of people living with HIV who have these conditions are left untreated [4–7, 34], in part because of significant fragmentation in the integration of mental health and addiction health care [24, 35–38]. Third, the current HIV care model focuses on treating HIV-related conditions and managing cART [39]. Bess and colleagues examined practices of depression treatment among 72 HIV health care providers [40]. The researchers found that only 31% of HIV providers would regularly screen their patients for depression, and only 13% of HIV providers would follow-up with the patients after prescribing antidepressants [40]. Additionally, Curran et al. (2011) found a number of key patient-, provider-, and system-level challenges confronting HIV health care providers in providing mental health care, including: time constraints; concerns regarding drug interactions between cART and antidepressants; difficulties with referrals to mental health specialists; overlap between depression and HIV symptoms; and inadequate expertise in treating depression [41]. These factors might increase the use of potentially avoidable ED encounters.

Our findings suggest that there is an urgent need to reform the current HIV care model in order to effectively manage multiple mental and physical co-morbidities [42, 43]. Several recent meta-analyses have suggested that integrated or collaborative care models with treatments that directly address mental health problems would improve health outcomes and quality of life in patients with cancer or diabetes [44–48]. A recent meta-analysis indicated that improving coordination of care (e.g., multi-disciplinary team models, decision support systems, case management or self-management strategies, etc.) might reduce the use of acute care among patients with multiple physical and mental co-morbidities [49]. Specifically for people living with HIV, a recent U.S. study shows that a collaborative depression care model is effective in improving quality-of-life and saving costs [50].

Several limitations in the current study need to be acknowledged. First, the screening instruments used to identify current depression could lead to false positives. This bias is likely to be non-differential and might drive our study associations towards null. Similarly, we relied on self-reported data to identify recreational drug use; misclassification is possible, and we were limited by not having information on the frequency or intensity of recreational drug use. Second, our HIV-positive participants were recruited from HIV speciality clinics, where they might receive more regular and better care than other people living with HIV. These patients might be less likely to use acute care services, so our results might not generalize to the larger HIV-positive population in Ontario. Third, we did not consider other important health system-level and personal health choice factors that might influence acute care services use. Future research should evaluate the potential influence of these factors and contexts.

## Conclusions

We found a significant increase in ED services use and potentially avoidable ED encounters (including low-acuity or repeated ED encounters), particularly among those with either current depression or recreational drug use. These findings emphasize the challenges of managing HIV and mental health and addiction co-morbidities in the current HIV care model. Future research is needed to examine and evaluate integrated, collaborative care programs and other strategies to improve coordination of care to effectively recognize and treat mental health and addiction problems among people living with HIV in Ontario and Canada.

Our results support the direction of Ontario’s HIV/AIDS Strategy to 2026, which addresses social and medical concerns associated with HIV (such as depression and substance use) and the social drivers of health in order to enhance the overall well-being of people living with or at risk of HIV. Our findings reinforce the importance of providing effective (and proactive) mental health screening and care (as well as access and continuity of service). They also demonstrate the need for longer term support and routine management of depression and substance use, particularly for those individuals at highest risk, to minimize the unintended use of more expensive acute care services in Ontario.

## Supporting information

S1 Table Full multivariate models for association between depression and recreational drug use exposure and two acute care services use outcomes (N = 3,482). (DOCX)

S2 Table Full multivariate model for associations between current depression and recreational drug use exposure and three potentially avoidable emergency department (ED) encounter outcomes (N = 2,108). (DOCX)
