# Supplementary material for: Impact of depression and recreational drug use on emergency department encounters and hospital admissions among people living with HIV in Ontario: A secondary analysis using the OHTN cohort study
Source: PLoS One. 2018 Apr 9;13(4):e0195185. doi: 10.1371/journal.pone.0195185 (PMC5891004; doi:10.1371/journal.pone.0195185)
Supplement: S1 Table — (DOCX) [file pone.0195185.s001.docx]

**S1 Table. Full Multivariate Models for Association between Depression and Recreational Drug Use Exposure and Two Acute Care Services Use Outcomes (N=3,482)**

| **Characteristics** | **Emergency Department Encounters** | | **Hospital Admissions** | |
| --- | --- | --- | --- | --- |
|  | **aHR** | **95% CI** | **aHR** | **95% CI** |
| **Main Exposure** |  |  |  |  |
| Depression-only ^f^ | **1.20** | **(1.06, 1.36)** | **1.27** | **(1.06, 1.53)** |
| Recreational drug use--only ^g^ | **1.33** | **(1.14, 1.55)** | 1.14 | (0.88, 1.48) |
| Co-occurring depression and recreational drug use | **1.44** | **(1.19, 1.74)** | **1.56** | **(1.19, 2.05)** |
| Without depression and recreational drug use (reference) | 1 |  | 1 |  |
|  |  |  |  |  |
| **Need** |  |  |  |  |
| History of depression ^a^ |  |  |  |  |
| Yes | **1.16** | **(1.05, 1.28)** | **1.21** | **(1.04, 1.41)** |
| No (reference) | 1 |  | 1 |  |
| History of drug addiction/dependence ^b^ |  |  |  |  |
| Yes | **1.28** | **(1.14, 1.45)** | -- | -- |
| No (reference) | 1 |  | -- | -- |
| Antidepressant use ^c^ |  |  |  |  |
| Yes | -- | -- | **0.81** | **(0.68, 0.95)** |
| No (reference) | -- | -- | 1 |  |
| Physical component of SF-12 (Increased by every five points) | **1.07** | **(1.05, 1.10)** | **1.18** | **(1.15, 1.22)** |
| Charlson multi-morbidity index ≥ 1 |  |  |  |  |
| Yes | **1.37** | **(1.23, 1.52)** | **1.23** | **(1.04, 1.46)** |
| No (reference) | 1 |  | 1 |  |
| Non-suppressed recent viral loads (> 50$\mu L$) (in past 6 months) |  |  |  |  |
| Yes | **1.17** | **(1.04, 1.31)** | **1.30** | **(1.10, 1.56)** |
| No (reference) | 1 |  | 1 |  |
| CD4 cell counts (<200 $\mu L$) (in past 6 months) |  |  |  |  |
| Yes | **1.19** | **(1.02 ,1.39)** | **1.77** | **(1.45, 2.17)** |
| No (reference) | 1 |  | 1 |  |
| Years since HIV diagnosis (Increased by every year) | -- | -- | **1.02** | **(1.01, 1.03)** |
|  |  |  |  |  |
| **Predisposing** |  |  |  |  |
| Age |  |  |  |  |
| 16-29 years | **1.52** | **(1.22, 1.90)** | 0.84 | (0.58, 1.26) |
| 30-39 years | 1.13 | (0.98, 1.31) | 0.85 | (0.67, 1.09) |
| 40-49 years | 0.97 | (0.87, 1.09) | **0.74** | **(0.62, 0.87)** |
| ≥ 50 years (reference) | 1 |  | 1 |  |
| Gender |  |  |  |  |
| Female | 0.89 | (0.76, 1.04) | -- | -- |
| Male (reference) | 1 |  | -- |  |
| Sexual orientation |  |  |  |  |
| Gay, lesbian, or bisexual | **0.78** | **(0.69, 0.88)** | **0.76** | **(0.65, 0.89)** |
| Heterosexual (reference) | 1 |  | 1 |  |
| Marital status |  |  |  |  |
| Married / living with partner | 1.11 | (1.00, 1.23) | -- | -- |
| Single, separated/divorced, or widowed (reference) | 1 |  | -- | -- |
| Ethnic identity |  |  |  |  |
| First Nation, Metis, or Inuit | 1.16 | (1.00, 1.35) | 1.08 | (0.86, 1.36) |
| African, Caribbean, Asian, or Latin American | 0.89 | (0.78, 1.01) | **0.74** | **(0.60, 0.91)** |
| European descent (reference) | 1 |  | 1 |  |
| Current employment status |  |  |  |  |
| Unemployed | 1.04 | (0.86, 1.26) | 1.33 | (1.00, 1.77) |
| Student/retired | **1.21** | **(1.01, 1.44)** | **1.75** | **(1.37, 2.34)** |
| Recipient of Ontario Disability Support Program | 1.11 | (0.98, 1.27) | **1.27** | **(1.06, 1.53)** |
| Employed (reference) | 1 |  | 1 |  |
|  |  |  |  |  |
| **Enabling** |  |  |  |  |
| Annual household income (CAD) before withholding taxes/benefits |  |  |  |  |
| < $20,000 | **1.19** | **(1.02, 1.40)** | -- | -- |
| $20,000 to $39,999 | **1.23** | **(1.07, 1.42)** | -- | -- |
| $40,000 to $49,999 | **1.25** | **(1.07, 1.45)** | -- | -- |
| ≥ $50,000 (reference) | 1 |  | -- | -- |
| Difficulty in affording housing-related expenses ^d^ |  |  |  |  |
| Yes | 1.01 | (0.90, 1.14) | -- | -- |
| No (reference) | 1 |  | -- | -- |
| Worry about eviction ^e^ |  |  |  |  |
| Yes | **1.18** | **(1.04, 1.35)** | -- | -- |
| No (reference) | 1 |  | -- | -- |
|  |  |  |  |  |
| **Instrument type** ^f^ |  |  |  |  |
| K_10_ | 1.01 | (0.91, 1.11) | 0.88 | (0.76, 1.02) |
| CES-D_20_ (reference) | 1 |  | 1 |  |

This table contains the final set of covariates retained in the multivariable Cox proportional hazard regression models for the index emergency department encounters and hospital admissions.

aHR = Adjusted hazard ratios

CI = Confidence intervals

^a^ History of depression was defined as having a past depression-related diagnosis in OHIP records (OHIP ICD-9: 296 and 311), from the earliest available records to a year before baseline.

^b^ History of drug addiction/dependence was defined as a diagnostic code of drug dependence/addiction in OHIP (ICD-9: 304) from the earliest available records to a year before the baseline.

^c^ The definition of antidepressants was based on the first line of antidepressants for managing depression in adults recommended by the Canadian Network for Mood and Anxiety Treatments (CANMAT) Clinical guidelines (Lam et al., 2009)

^d^ Difficulty in affording house-related expenses was defined as a patient’s self-reported “Very difficult” or “Fairly difficult” to the following question: *“Considering your household income, how difficult is it for you to meet your monthly housing-related costs?(Housing costs include rent/mortgage, property taxes and utilities only).”*

^e^ A 5-point Likert scale (strongly agree to strongly disagree) was used. We dichotomized their response into “yes” (strongly agree/agree) and “no” (neutral/disagree/strongly disagree).

^f^ There are two instruments for identifying current depression administered by clinic nurses and assistants during the participant’s regular clinical appointments. Due to constraints on human resources and time in several HIV clinics, 61% of HIV-positive participants were administered the 10-item Kessler Psychological Distress Scale (K_10_) and 39% were administrated the 20-item Centre for Epidemiologic Studies Depression Scale (CES-D_20_). Full details of the cohort can be found on the study website: <http://www.ohtncohortstudy.ca/>

^g^ Participants were asked whether they had used any of the following drugs for recreational or other non-medical purposes over the past six months: anabolic steroids, amphetamines, methamphetamines, cocaine, crack/freebase, club drugs, heroin, opiates, tranquilizers, or other substances.
