# Supplementary material for: Impact of depression and recreational drug use on emergency department encounters and hospital admissions among people living with HIV in Ontario: A secondary analysis using the OHTN cohort study
Source: PLoS One. 2018 Apr 9;13(4):e0195185. doi: 10.1371/journal.pone.0195185 (PMC5891004; doi:10.1371/journal.pone.0195185)
Supplement: S2 Table — (DOCX) [file pone.0195185.s002.docx]

**S2 Table. Full Multivariate Model for Associations between Current Depression and Recreational Drug Use Exposure and Three Potentially Avoidable Emergency Department (ED) Encounter Outcomes (N=2,108)**

| **Characteristics** | **Low-acuity ED**  **Encounters ^a^** | | **Repeated ED Encounters ^b^** | | **ED encounters without seeking for ambulatory care 30 days prior to the index encounters ^c^** | |
| --- | --- | --- | --- | --- | --- | --- |
|  | **aHR** | **95% CI** | **aHR** | **95% CI** | **aHR** | **95% CI** |
| **Main Exposure** |  |  |  |  |  |  |
| Depression-only ^g^ | **1.27** | **(1.07, 1.50)** | **1.27** | **(1.03, 1.55)** | 0.97 | (0.77, 1.25) |
| Recreational drug use--only ^h^ | **1.28** | **(1.04, 1.58)** | **1.46** | **(1.13, 1.87)** | **1.52** | **(1.17, 1.99)** |
| Co-occurring depression and recreational drug use | **1.32** | **(1.04, 1.67)** | **1.42** | **(1.06, 1.89)** | 0.94 | (0.65, 1.35) |
| Without depression and recreational drug use (reference) | 1 |  | 1 |  | 1 |  |
|  |  |  |  |  |  |  |
| **Need** |  |  |  |  |  |  |
| History of drug addiction/dependence ^d^ |  |  |  |  |  |  |
| Yes | -- | -- | **1.33** | **(1.11, 1.66)** | -- | -- |
| No (reference) | -- | -- | 1 |  | -- | -- |
| Antidepressant use ^e^ |  |  |  |  |  |  |
| Yes | **0.73** | **(0.62, 0.85)** | **0.70** | **(0.58, 0.84)** | 0.84 | (0.68, 1.04) |
| No (reference) | 1 |  | 1 |  | 1 |  |
| Physical component of SF-12 (Increased by every five  points) | 1.00 | (0.97, 1.03) | **1.05** | **(1.01, 1.10)** | 1.01 | (0.96, 1.06) |
| Charlson multi-morbidity index ≥ 1 |  |  |  |  |  |  |
| Yes | -- | -- | **1.33** | **(1.12, 1.59)** | -- | -- |
| No (reference) | -- | -- | 1 |  | -- | -- |
| CD4 cell counts (<200 $\mu L$) (in past 6 months) |  |  |  |  |  |  |
| Yes | -- | -- | **1.32** | **(1.05, 1.60)** | **1.36** | **(1.03, 1.79)** |
| No (reference) | -- | -- | 1 |  | 1 |  |
| Years since HIV diagnosis (increased by every year) | -- | -- | **0.99** | **(0.97, 0.998)** | 0.99 | (0.98, 1.01) |
|  |  |  |  |  |  |  |
| **Predisposing** |  |  |  |  |  |  |
| Age |  |  |  |  |  |  |
| 16-29 years | -- | -- | -- | -- | **2.19** | **(1.49, 3.24)** |
| 30-39 years | -- | -- | -- | -- | 1.24 | (0.92, 1.67) |
| 40-49 years | -- | -- | -- | -- | **1.29** | **(1.03, 1.61)** |
| ≥ 50 years (reference) | ***--*** | ***--*** | -- | -- |  |  |
| Gender |  |  |  |  |  |  |
| Female | 0.88 | (0.72, 1.08) | 0.92 | (0.73, 1.15) | 0.78 | (0.58, 1.03) |
| Male (reference) | 1 |  | 1 |  | 1 |  |
| Sexual orientation |  |  |  |  |  |  |
| Gay, lesbian, or bisexual | **0.78** | **(0.67, 0.92)** | -- | -- | **0.74** | **(0.59, 0.93)** |
| Heterosexual (reference) | 1 |  | -- | -- | **1** |  |
| Immigration status |  |  |  |  |  |  |
| Canadian immigrant | **0.77** | **(0.65, 0.91)** | 0.82 | (0.67, 1.00) | -- | -- |
| Canadian born (reference) | 1 |  | 1 |  | -- | -- |
| Current employment status |  |  |  |  |  |  |
| Unemployed | 1.17 | (0.91, 1.49) | 1.35 | (1.00, 1.83) | 0.92 | (0.65, 1.31) |
| Student/retired | 1.00 | (0.78, 1.29) | 1.22 | (0.90, 1.66) | 0.95 | (0.66, 1.35) |
| Recipient of Ontario Disability Support Program | 1.07 | (0.89, 1.28) | 1.21 | (0.97, 1.50) | 0.79 | (0.62, 1.00) |
| Employed (reference) | 1 |  | 1 |  | 1 |  |
| Education attainment |  |  |  |  |  |  |
| Completed high school or less | -- | -- | -- | -- | **1.23** | **(1.01, 1.50)** |
| More than high school (reference) | -- | -- | -- | -- | **1** |  |
|  |  |  |  |  |  |  |
| **Enabling** |  |  |  |  |  |  |
| Annual household income (CAD) before withholding  taxes/benefits |  |  |  |  |  |  |
| < $20,000 | **1.32** | **(1.08, 1.60)** | **1.41** | **(1.11, 1.80)** | **1.50** | **(1.13, 1.99)** |
| $20,000 to $39,999 | 1.16 | (0.95, 1.41) | 1.13 | (0.87, 1.45) | 1.30 | (0.98, 1.71) |
| $40,000 to $49,999 | 1.03 | (0.83, 1.28) | **1.33** | **(1.02, 1.72)** | **1.41** | **(1.05, 1.89)** |
| ≥ $50,000 (reference) | 1 |  | 1 |  | 1 |  |
| Difficulty in affording housing-related expenses ^f^ |  |  |  |  |  |  |
| Yes | 0.99 | (0.84, 1.16) | 0.94 | (0.77, 1.14) | 1.09 | (0.87, 1.36) |
| No (reference) | -- | -- | 1 |  | 1 |  |
|  |  |  |  |  |  |  |
| **Instrument type** ^g^ |  |  |  |  |  |  |
| K_10_ | **1.21** | **(1.05, 1.39)** | 0.93 | (0.78, 1.10) | 1.16 | (0.96, 1.40) |
| CES-D_20_ (reference) | 1 |  | 1 |  | 1 |  |

This table contains the final set of covariates retained in the multivariable Cox proportional hazard regression models for the three potentially avoidable emergency department encounter outcomes.

aHR = Adjusted hazard ratios

CI = Confidence intervals

^a^ Low-acuity ED encounters were identified using level four or five of the five-level Canadian triage and acuity scale (1=resuscitation, 2=emergency, 3=urgent, 4=semi-urgent, 5=non-urgent) AND a visit disposition at discharge that indicated that participants were not transferred to inpatient care.

^b^ Repeated ED encounters were defined by discharge date of an emergency department encounter that occurred within 30 days of a previous encounter at the same or a different facility.

^c^ Emergency department encounters without seeking ambulatory care were defined as emergency department encounters without an ambulatory visit to a physician in the 30 days prior to the index encounter.

^d^ History of alcoholism was defined as a diagnostic code of alcohol dependence/abuse in OHIP (ICD-9: 303) from the earliest available records to a year before the baseline.

^e^ The definition of antidepressants was based on the first line of antidepressants for managing depression in adults recommended by the Canadian Network for Mood and Anxiety Treatments (CANMAT) Clinical guidelines (Lam et al., 2009)

^f^ Difficulty in affording house-related expenses was defined as a patient’s self-reported “Very difficult” or “Fairly difficult” to the following question: *“Considering your household income, how difficult is it for you to meet your monthly housing-related costs?(Housing costs include rent/mortgage, property taxes and utilities only).”*

^g^ There are two instruments for identifying current depression administered by clinic nurses and assistants during the participant’s regular clinical appointments. Due to constraints on human resources and time in several HIV clinics, 61% of HIV-positive participants were administered the 10-item Kessler Psychological Distress Scale (K_10_) and 39% were administrated the 20-item Centre for Epidemiologic Studies Depression Scale (CES-D_20_). Full details of the cohort can be found on the study website: <http://www.ohtncohortstudy.ca/>

^h^ Participants were asked whether they had used any of the following drugs for recreational or other non-medical purposes over the past six months: anabolic steroids, amphetamines, methamphetamines, cocaine, crack/freebase, club drugs, heroin, opiates, tranquilizers, or other substances
